# Prevalence of Noncardiac Findings in Patients Undergoing Cardiac Magnetic Resonance Imaging

**DOI:** 10.1100/2012/474582

**Published:** 2012-04-01

**Authors:** Christopher W. May, William T. Mansfield, Andrew B. Landes, Adrian M. Moran

**Affiliations:** ^1^Department of Cardiology, Maine Medical Center, 88 Beamhall st, Portland, ME 04102, USA; ^2^Department of Radiology, Maine Medical Center, 88 Beamhall st, Portland, ME 04102, USA; ^3^Pediatric Division of Cardiology, Department of Pediatrics, 887 Congress Street, Suite 310, Portland, ME 04102, USA

## Abstract

*Purpose*. We sought to determine the prevalence of clinically significant non-cardiac abnormalities found in pediatric and adult patients undergoing cardiac magnetic resonance imaging (CMRI), and understand the impact of age on it's occurrence. *Methods*. We retrospectively reviewed all patients undergoing CMRI between May 2004 and July 2007. Findings were considered significant if they required radiographic or clinical follow-up. *Results*. A total of 408 patients underwent CMRI during the study period. Twenty two (16%) pediatric patients (age < 19 years, *n* = 135) were found to have a total of 22 non- cardiac abnormalities, 3 of which were clinically significant. Sixty four (23%) adult patients (age > 19 years, *n* = 273) were found to have a total of 77 non-cardiac abnormalities, 33 of which were clinically significant. The prevalence of clinically significant non-cardiac abnormalities was 2% in the pediatric cohort and 11% in the adult cohort (*P* = 0.05). Within the adult population, the prevalence of significant non-cardiac abnormalities increased with advancing age (*P* = 0.05). *Conclusions*. In a population of unselected patients undergoing CMRI, unanticipated noncardiac abnormalities were frequently seen. A small number of these were significant, with the prevalence increasing with age.

## 1. Introduction

CMRI is an evolving technology for noninvasive imaging of the heart, pericardium, and great vessels with increasing application in clinical cardiology. The high temporal and spatial resolution of CMRI affords versatility and potential superiority over other modalities for imaging patients with complex congenital heart disease, cardiomyopathies, and valvular pathology [1, 2]. Currently, as recognized by the American College of Cardiology Foundation, there are seventeen indications considered appropriate for CMRI, divided into the three broad categories consisting of coronary artery disease, cardiac structure and function, and myocardial viability [3]. As utilization of CMRI accelerates, so does the probability of detecting noncardiac abnormalities within the thorax, mediastinum, and abdomen, in part because of the expanded field of view afforded. Currently, little is known of the prevalence of noncardiac abnormalities found in patients of varying ages undergoing CMRI with three studies suggesting a prevalence of between 7.6% and 43% [4–6]. Therefore, we sought to determine the prevalence of clinically significant noncardiac abnormalities found in patients undergoing CMRI by performing a retrospective review of all CMRI studies performed in pediatric and adult patients at our institution between July 2004 and July 2007.

## 2. Methods

### 2.1. Patient Population

The study group consisted of all patients who underwent cardiac magnetic resonance imaging at Maine Medical Center (Portland, ME) between July 2004 and July 2007. Patients were primarily referred for CMRI by either a pediatric cardiologist or their primary adult cardiologist, with the majority of patients being ambulatory at the time of their CMRI. Reports were retrospectively reviewed, with all abnormalities involving noncardiac structures identified and categorized according to organ system and clinical significance. Abnormal noncardiac findings involved structures other than the heart, pericardium, and great vessels and were included in the study if they were unknown or unanticipated by the readers at the time of interpretation. Noncardiac findings were considered significant if additional radiographic imaging was advised or if further clinical investigation was warranted [5]. Recommendations by the readers for additional radiographic followup adhered to practice guidelines; examples included followup for mediastinal lymphadenopathy (LAd) greater than 1 centimeter, masses (e.g., other than simple cysts), pleural effusions, and vertebral abnormalities. Followup of patients with clinically significant noncardiac abnormalities was verified by reviewing radiographic studies and medical records available within our institution. Because of the potential differences in indications for CMRI, as well as age-related differences in comorbidities, the study population was divided into a pediatric cohort (age ≤ 19 years) and an adult cohort (age ≥ 19 years). Institutional review board approval was obtained prior to initiation of the study.

### 2.2. Data Acquisition

Images were obtained on a 1.5 Tesla GE (GE Healthcare, Milwaukee, Wis) magnetic resonance scanner using a cardiac 8-element phased-array coil and dedicated cardiac software. Standardized imaging protocols included black blood double inversion recovery sequences (DIRV), steady state free precession (SSFP) and fast gradient echo images, phase velocity flow mapping, magnetic resonance angiography, and postgadolinium myocardial delayed enhancement. Imaging protocols were determined by clinical indication and reviewed by the interpreting physician at the time of acquisition. Axial imaging was performed to include the entire chest from aortic arch to below the diaphragm using a single-phase SSFP localizer scan and a DIRV sequence, with cine sequences performed with smaller FOV's centered on the heart frequently tailored to the specific clinical question. All studies were jointly interpreted by a cardiologist (AMM) and a radiologist (ABL), with the creation of a single report which was stored in an electronic database, PACS Web1000 (AGFA, Mortsel, Belgium).

### 2.3. Statistical Analysis

To determine whether the prevalence of noncardiac findings differed with age, we divided the adult cohort into age blocks of twenty years. Comparisons were made between the pediatric and adult cohorts, as well as between each of the adult age blocks. Due to the small number of patients in the age block of 80 years and older, this age block was not included in the analysis. Age category comparative analysis was conducted with the *χ* ^2^ test for independence, using one degree of freedom at the *P* = 0.05 level of significance. Fischer's exact test was not required as the expected frequencies were greater than 5 for each of the comparisons made.

## 3. Results

### 3.1. Demographics

A total of 408 consecutive patients underwent CMRI at our institution during the study period. The study group was comprised of 210 males and 198 females, with a median age of 30 years (range 6 weeks to 85 years). There were 135 patients in the pediatric cohort, with a median age of 14 years (range 6 weeks to 19 years). Within this cohort, the most common clinical indication for CMRI was complex congenital heart disease (38%), followed by evaluation for arrhythmogenic right ventricular dysplasia (ARVD) (21%) and aortic disease, including aortic valve pathology (17%). In the adult cohort, there were a total of 273 patients, with a median age of 45 years (range, 20–85 yrs). The most common clinical indication for CMRI in the adult cohort was evaluation for ARVD (31%), followed by complex congenital heart disease (25%) and cardiomyopathy (13%) (Table 1).

### 3.2. Pediatric Cohort

Twenty-two of the 135 patients (16%) were found to have a total of 22 noncardiac abnormalities. Three of these 22 noncardiac abnormalities (14%) were considered clinically significant. There was no dominant category of clinically significant abnormality nor was one organ system more likely to have a clinically significant abnormality than another. Overall, 2% of the pediatric cohort was found to have clinically significant noncardiac abnormalities on CMRI (Table 2).

**Figure 1:**
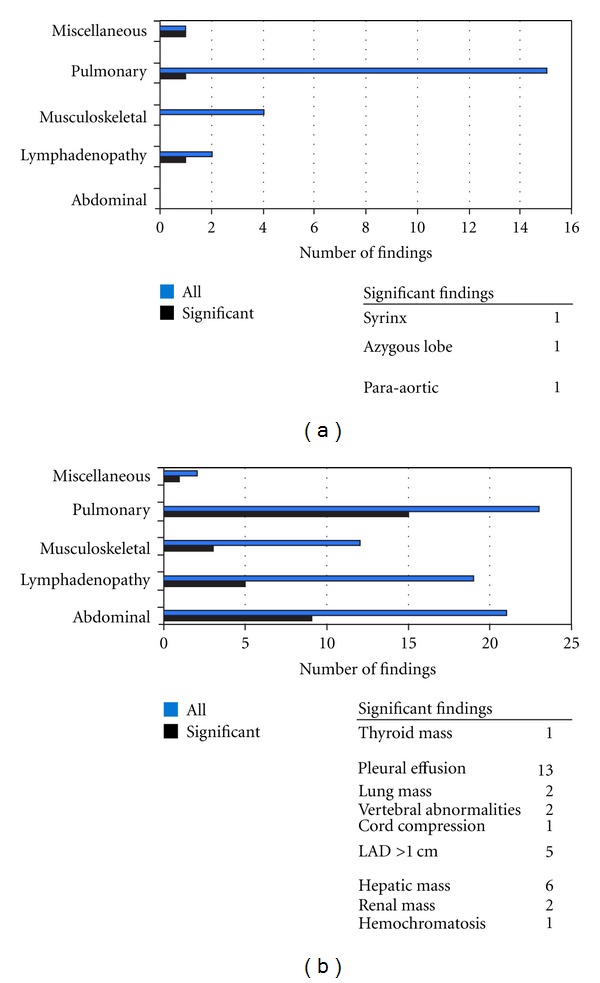
Organ involvement of noncardiac findings in the pediatric and adults cohorts.

Organ system involvement of the noncardiac abnormalities is depicted in Figure 1(a). The three clinically significant noncardiac abnormalities consisted of para-aortic LAd > 1 cm, suspected azygous lobe of the right lung and syringomyelia. The patient with para-aortic LAd had a history of non-Hodgkin's lymphoma; comparison of the CMRI with a computed tomography (CT) obtained three months earlier showed stability of the LAd. Long-term radiographic followup was recommended to monitor recurrence of disease. One patient had a low-intensity signal adjacent to the trachea and superior vena cava (SVC), and a chest X-ray was recommended to determine if this abnormality was an azygous lobe of the right lung. The patient with suspected syringomyelia underwent magnetic resonance imaging (MRI) of the thoracic and lumber spine two months later, confirming the diagnosis. There was no evidence of spinal cord compromise, and the patient had an additional MRI six months later for continued monitoring.

Among the noncardiac abnormalities not requiring followup, pulmonary findings comprised the largest group (*n* = 15), representing 68% of all noncardiac abnormalities. Ten of the pulmonary abnormalities were in patients who were intubated at the time of CMRI acquisition and included atelectasis (*n* = 6), pleural effusions (*n* = 2), consolidation (*n* = 1), and a collapsed lung (*n* = 1). No followup was recommended for these 10 findings. The remaining noncardiac abnormalities not requiring followup were airway narrowing without evidence of mass effect (*n* = 4), scoliosis (*n* = 4), and subcentimeter mediastinal LAd (*n* = 1).

### 3.3. Adult Cohort

Sixty-four of the 273 patients (23%) were found to have a total of 77 noncardiac abnormalities. Thirty-three of these 77 noncardiac abnormalities (43%) were considered clinically significant. While LAd, pulmonary abnormalities, and abdominal abnormalities were relatively equal in comprising the overall number of noncardiac abnormalities, pulmonary abnormalities represented nearly half of the clinically significant findings. Overall, 11% of the adult cohort was found to have clinically significant noncardiac abnormalities on CMRI (Table 2).

Organ system involvement of the noncardiac abnormalities is depicted in Figure 1(b). Clinically significant pulmonary abnormalities included pleural effusions (*n* = 13) and lung masses (*n* = 2). All of the patients with pleural effusions had been hospitalized prior to the CMRI and had radiographic and clinical followup for the persistence of the effusions. Two of the patients with pleural effusions underwent diagnostic thoracentesis as part of their clinical followup, and all thirteen patients were eventually diagnosed with decompensated heart failure. The two patients with lung masses had prior diagnoses relevant to these findings: one patient had a prior history of nonsmall cell lung cancer while the other patient was known to have sarcoidosis. Though the clinical diagnosis for each patient was documented in the medical record, the readers had to compare the CMRI to prior films, and serial radiographic studies were suggested for each of the patients. Pulmonary findings not requiring followup included airway narrowing without evidence of mass effect (*n* = 3), pulmonary hypoplasia (*n* = 2), atelectasis (*n* = 2), and pneumonectomy in a patient with a history of mediastinal teratoma resection.

There were twenty patients with 21 abdominal abnormalities, 16 of which were either renal or hepatic masses. Nine of the abdominal findings (43%) were clinically significant. Six of the hepatic lesions (75%) and two of the renal lesions (25%) required additional imaging to exclude malignancy or to determine if the finding was a simple cyst. The remaining clinically significant abdominal abnormality involved one patient, whose liver on T1-weighted imaging appeared lower intensity than spleen and muscle, raising the suspicion of hemochromatosis; additional investigation was recommended. None of the seven patients with the clinically significant abdominal masses was diagnosed with a malignancy. Abdominal findings not requiring followup included ascites in a patient with known cirrhosis, hiatal hernias (*n* = 2), and an accessory spleen. The category of abdominal abnormalities was the only one in which there was a difference among readers' interpretations; for two patients, the radiologist amended the report to clarify the identity of the noncardiac abnormality.

There were 19 patients with LAd, though only 5 (20%) patients had LAd >1 cm requiring additional radiographic followup (Table 2). Among the five patients, one had surveillance imaging for greater than one year while another had mediastinoscopy with biopsy to exclude malignancy. None of these patients were found to have malignancies. Granulomatous disease was the most common final diagnosis (*n* = 2).

The incidence of clinically significant vertebral abnormalities was 25%  (*n* = 3), similar to that of clinically significant LAd. One patient was thought to have an expansile lesion, one was found to have a lytic lesion of thoracic vertebra 12, and one patient had severe degenerative disc disease with herniation and suspected of cord compression. Vertebral abnormalities not requiring followup included scoliosis (*n* = 6), vertebral hemangiomas (*n* = 3), and severe degenerative changes of the spine (*n* = 1).

The two miscellaneous noncardiac abnormalities were comprised of a patient with bilateral breast implants and one patient with a thyroid mass. The patient with the thyroid mass underwent further imaging and was diagnosed with a calcified cyst.

**Figure 2:**
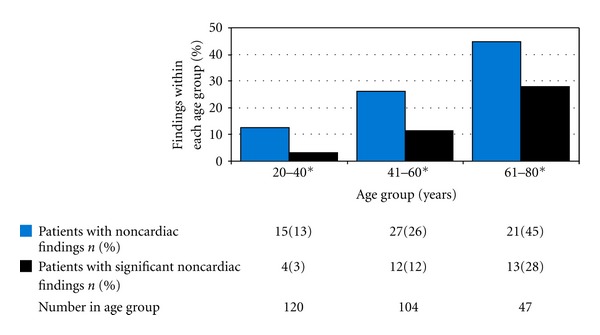
Prevalence of noncardiac findings by age within adult cohort. The difference in the percent of significant noncardiac findings between these age groups was statistically significant (*P* < 0.05 using the *χ* ^2^ test for independence). The age group greater than 80 years of age was comprised of one patient with a noncardiac finding which was not significant. This age group was not included in our analysis due to the small sample size.

Comparing the pediatric and adult cohorts, the prevalence of significant noncardiac findings was statistically significant (2% versus 11%, *P* = 0.05). This finding was due to the increasing prevalence of significant noncardiac findings in patients older than forty years of age (Figure 2).

## 4. Discussion

Our study is the first to report the prevalence of noncardiac abnormalities in a series of unselected consecutive patients across a broad age range undergoing CMRI at a single institution. We retrospectively reviewed 408 patients who underwent CMRI, dividing the study population into pediatric and adult cohorts based on age. Within the pediatric cohort, 16% of the patients were found to have noncardiac abnormalities, with 14% of these abnormalities considered clinically significant and requiring additional followup. In the adult cohort, 23% of patients were found to have noncardiac abnormalities, with 43% of the findings considered clinically significant. Overall, 2% of the pediatric cohort and 11% of the adult cohort had noncardiac abnormalities found on CMRI that were considered clinically significant. To our knowledge, this is the first to report such findings in both pediatric and adult patients and determine the impact of increasing age on prevalence of noncardiac findings.

We divided the study population into pediatric and adult cohorts given the unselected population, the differences in CMRI indication, and the greater potential for comorbidities commonly seen with advancing age. Pediatric patients were more likely to undergo CMRI for complex congenital heart disease and evaluation of the aortic valve and arch, while adult patients were more likely to be evaluated for ARVD and myocardial scar. In the pediatric cohort, pulmonary abnormalities comprised two-thirds of total noncardiac findings. In the adult cohort, LAd, pulmonary, and abdominal abnormalities were approximately equal in number and collectively represented 83% of all noncardiac findings. There was a marked variety in the breath of noncardiac findings detected by CMRI, with clinically significant abnormalities ranging from pleural effusions, LAd, and abdominal masses to syringomyelia, vertebral lesions, possible spinal cord compression, and a thyroid mass. Although 39 patients (2 pediatric, 37 adults) had either LAd or a mass detected on CMRI, no new malignancies were diagnosed in the study population.

To date no study has assessed the prevalence of noncardiac findings across a broad pediatric and adult age range. Chan et al. [4] reported on 1534 patients (average age 50 years) performed at an academic center over a 5-year period. They found 7.6% of studies with noncardiac findings consisting of major findings in 3.4% (adenopathy, lung abnormalities, and mediastinal masses) and 4.6% with minor abnormalities. They noted that the majority (62%) of the major findings were previously known, with only 0.4% of the findings deemed new and clinically important. They found that the average age was higher in those with associated noncardiac findings. Dewey et al. studied 108 adult patients with suspected coronary artery disease (CAD) who underwent CMRI and CCTA prior to diagnostic cardiac catheterization, comparing the rate of significant and insignificant noncardiac findings for both of the noninvasive imaging modalities [5]. Imaging protocols were based on the intended objective of evaluating the patients for CAD. A total of 16 noncardiac findings (15%) were detected by CCTA, of which five were considered significant. These five abnormalities consisted of pulmonary embolism, pleural effusions, sarcoidosis, a pulmonary nodule, and a hiatal hernia. CMRI detected nine noncardiac abnormalities (8%); only two of the findings (pleural effusions and sarcoidosis) were considered significant. Overall, 5% and 2% of patients were found to have clinically significant noncardiac abnormalities by CCTA and CMRI, respectively. More recently, Atalay et al. [6] reported on 240 CMRI examinations that included 8 pediatric patients. They reported a rate of 43% with noncardiac findings with a higher occurrence in older patients (>60 years) (43% versus 17%). The distribution of findings was 29% in the abdomen, 70% in the chest, and 1% in the neck. Of note, in their study, five new cases of cancer were diagnosed. This regional distribution of noncardiac findings was similar to our adult subgroup (0.03% neck, 70% chest, and 27% abdomen).

Recognizing the prevalence of noncardiac abnormalities on cardiac imaging studies is important for the ordering and interpreting physicians, as well as for the patient. This is particularly relevant at a time when clinical utilization of CMRI and CCTA is increasing. Comparing the prevalence of noncardiac findings on CMRI and CCTA is a reasonable endeavor, given that both modalities offer high spatial and temporal resolution, are diagnostic, and have the ability to detect abnormalities within an expanded FOV. Substantially more data is available regarding the prevalence of noncardiac abnormalities detected by CCTA [5, 7–13], which ranges from 11% to 67% of patients (Table 3). The broad range in prevalence of noncardiac abnormalities most likely reflects the differences in imaging protocols, definitions of noncardiac findings, and the differences in FOV used in the CCTA studies.

CCTA utilizes a small FOV in order to maximize voxel resolution while minimizing radiation exposure of the patient. Reduction in the number of slices along the *z*-axis ensures that only a portion of the lung parenchyma will be visualized. While complete data in the *x*-*y* direction is obtained, this expanded FOV is not used in analyzing the coronary arteries and is excluded without additional reconstructions. The restricted FOV typical of CCTA includes roughly 10% of lung tissue [14]. In a study comparing the restricted and expanded FOVs afforded by CCTA with those obtained by noncardiac chest CT, Haller et al. demonstrated that the restricted CCTA FOV includes 35.5% of the total chest volume while the maximum expanded CCTA FOV includes only 70.3% of total chest volume [8]. Though the complete thorax is imaged on CMRI, visualization of structures depends upon the sequences used: adenopathy is best appreciated on T1 (nonfat saturated) sequences while cysts are best seen on T2 and postgadolinium T1 sequences. Both modalities allow manipulation of the acquired data and FOV, with the imaging sequences determined principally by the clinical question and partly by the physician's tolerance for detecting unintended or unwanted noncardiac abnormalities. Currently, there are no standardized protocols for either modality addressing the issue of imaging sequences and size of FOV. There is, however, a medicolegal and medicomoral obligation, with the interpreting physician responsible to the patient for all of the information available on the examination [15]. Performing additional reconstructions using an expanded FOV (CCTA) or including additional sequences (CMRI), while not the initial purpose of either modality, may be most consistent with the ethical principle of beneficence [16, 17].

Detection of significant noncardiac abnormalities with either CMRI or CCTA potentially impacts patient care. CT is superior for imaging lung parenchyma, and much has been written about the detection of lung abnormalities by CCTA, principally lung nodules and masses [14]. The results of our study extend this discussion beyond the exclusion of malignancy to include diverse findings such as syringomyelia, hemochromatosis, and spinal cord compression, all of which can have an important impact on a patient's health. While the absolute number of patients with significant noncardiac abnormalities and the type of abnormalities varies (due to differences in patient population, clinical indication, and technique), current data suggest that noncardiac abnormalities detected by either modality are consequential both in numbers and potential clinical consequence. The spectrum of abnormalities found in our study emphasizes the need for physicians to be methodical and thorough in their interpretive approach, as well as competent in the analysis of the entire data set, and not just the cardiac structures and function. Furthermore, vigilance for probable noncardiac findings is necessary.

Our study is limited by the incomplete followup data of our patient population. While each patient's record within our institution was reviewed, seven adult patients and one pediatric patient with a clinically significant noncardiac finding did not have followup studies within our EMR/PACs, and therefore the clinical impact of the noncardiac abnormalities is not fully known. Two patients were still receiving continued monitoring of the noncardiac abnormalities at the end of the study, and therefore a final diagnosis is not yet known. The intent of our study was to report the prevalence of noncardiac findings on CMRI and not focus on long-term followup. Our institution does not perform cardiac perfusion or viability studies with CMRI, which may select out a certain segment of patients appropriate for CMRI. We did not attempt to address the potential economic impact of the noncardiac findings or comment on the nature of the recommended followup.

In conclusion, CMRI is a powerful and versatile noninvasive imaging modality with increasing clinical utilization for the evaluation of patients with known or suspected CAD, structural heart disease including complex congenital disease, and myocardial viability. The expanded field of view and diagnostic quality of the images affords the probability of detecting a wide variety of unanticipated noncardiac abnormalities, the likelihood of which increases with age. Therefore, studies should be interpreted in a thorough and methodical fashion by competent physicians who are appropriately trained, to ensure that all imaged structures are appropriately evaluated. Collaboration between cardiology and radiology is advisable to ensure all structures are adequately evaluated.

## Figures and Tables

**Table 1:** Study population.

	Pediatric (%)	Adult (%)
*Demographics*		
* n*	135	273
Male	79 (59)	131 (48)
Female	56 (41)	142 (52)
Median age, years (range)	14 (6 wks-19)	45 (20–85)

*Study indication*		
Aortic valve and arch	23 (17)	29 (11)
ARVD	29 (21)	86 (31)
Cardiomyopathy	12 (9)	35 (13)
Congenital*	51 (38)	68 (25)
Pericardial	1 (1)	8 (3)
Valvular	14 (10)	29 (11)
Miscellaneous	5 (4)	18 (6)

*Excluding isolated aortic valve disease.

**Table 2:** Comparison of noncardiac findings in pediatric and adult cohorts.

Pediatric			Adult	
Number of patients	Percent of population		Number of patients	Percent of population
22	16	All	64	23
3	2*	Significant	30	11

*The difference in prevalence of significant noncardiac findings in the pediatric and adult cohorts was statistically significant (*P* < 0.05 using the *χ* ^2^ test for independence).

**Table 3:** CCT studies examining noncardiac findings.

Reference	Year	Indication for CCT	*n* (Male/Female)	Mean age (yrs)	Noncardiac findings (%)	Significant noncardiac findings (%)
Haller et al. [8]	2005	Suspected CAD	166 (123/43)	64	36 (21.6)	18 (10.8)
Onuma et al. [12]	2006	Suspected CAD	503 (382/121)	66 ± 10	346 (58.1)	114 (22.7)
Dewey et al. [5]	2007	Suspected CAD	108 (80/28)	63 ± 9	11 (10.1)	5 (4.6)
Gil et al. [7]	2007	Asymptomatic, Self-referred	258 (202/56)	54 ± 8	151 (56.2)	40 (15.5)*
Kirsch et al. [9]	2007	Suspected CAD	100 (68/32)	63 ± 15	67 (67)	25 (25)*
Mueller et al. [11]	2007	Post-operative CABG	259 (190/69)	61 ± 27	34 (13.1)	26 (10)*
Law et al. [10]	2008	Suspected CAD	295 (198/97)	56	51 (17.2)	46 (15.6)*
MacHaalany et al. [13]	2009	Suspected CAD	966 (535/431)	58 ± 16	401 (41.5)	80 (20)

*Using our definition of significant noncardiac finding.
